# Mechanism and Efficacy of Cu_2_O-Treated Fabric

**DOI:** 10.3390/antibiotics11111633

**Published:** 2022-11-16

**Authors:** Zachary Benmamoun, Trent Wyhopen, You Li, William A. Ducker

**Affiliations:** 1 Department of Chemical Engineering, Virginia Tech, Blacksburg, VA 24061, USA; 2 Center for Soft Matter and Biological Physics, Virginia Tech, Blacksburg, VA 24061, USA

**Keywords:** copper, facemask, antibacterial, MRSA, *Pseudomonas aeruginosa*, *Streptococcus pneumoniae*, drug resistant

## Abstract

Pathogenic bacteria can remain viable on fabrics for several days and therefore are a source of infection. Antimicrobial fabrics are a potential method of reducing such infections, and advances in antimicrobial fabrics can be enhanced by knowledge of how the fabric kills bacteria. Metal oxides have been considered and used as antimicrobial ingredients in self-sanitizing surfaces, including in clinical settings. In this work, we examine how the addition of cuprous oxide (Cu_2_O) particles to polypropylene fibers kills bacteria. First, we show that the addition of the Cu_2_O particles reduces the viability of common hospital pathogens, *Staphylococcus aureus*, *Pseudomonas aeruginosa*, and *Streptococcus pneumoniae,* by 99.9% after 30 min of contact with the treated polypropylene. Then, we demonstrate that the main killing effect is due to the drying of the bacteria onto the cuprous oxide particles. There is also a weaker effect due to free Cu^+^ ions that dissolve into the liquid. Other dissolved species were unimportant. Chelation of these Cu^+^ ions in soluble form or precipitation removes their antimicrobial activity.

## 1. Introduction

Hospital-acquired infections (HAIs) prior to COVID accounted for 1.7 million infections, about 10,000 deaths, and multiple billion dollars in healthcare costs per annum in the United States alone [1]. Self-disinfecting materials are an attractive option for reducing the transmission of pathogenic microbes and for reducing waste. For example, facemasks are used as personal protective equipment and have been widely employed during the COVID-19 pandemic. As effective as they are at filtering microbes, they do not kill them, and therefore used facemasks are a potential source of infection. Facemasks are known to become contaminated during the treatment of patients [2,3] and then become a source of infection for other patients, many of whom have increased susceptibility to infection due to their conditions. Antimicrobial surface coatings are known to decrease both the number of HAIs and the number of bacteria in the environment [4,5,6] and, therefore, could be used on facemasks and other fabrics in environments rich in pathogenic organisms.

In this study, we investigate the mechanism of a Cu_2_O-treated antimicrobial polypropylene fabric. By mechanism, we mean the action of the Cu_2_O that kills the cell, not the biological mechanism within the cell. The ultimate goal is to produce personal protective equipment (PPE) that (i) rapidly kills bacteria responsible for HAIs, and (ii) can be functional for long periods of use. Such a product could be used in healthcare settings to reduce the transmission of deadly diseases among both patients and the general public. An important additional benefit would be that fabrics could be used for a longer period of time, reducing the amount of waste generated in hospitals. 

Cuprous oxide is a well-known antimicrobial substance and has been used in self-disinfecting materials, as well as in antifouling agents and fungicides [7,8,9,10,11,12]. Cu_2_O is a popular option due to its excellent antimicrobial performance, low risk of bacteria developing resistance, and lack of toxicity to humans [13]. These factors combine to make Cu_2_O an attractive option for an antibacterial facemask. Cuprous oxide has been used frequently as an antimicrobial additive to textiles in research, as well as being used in an antiviral and antibacterial facemask in a study by Borkow et al. [14].

In this work, we present the antibacterial efficacy of Cu_2_O particles that were adhered to polypropylene nonwoven textiles via heat treatment. Microparticles rather than nanoparticles were used to avoid possible health issues with nanoparticles. In comparison to the prior work by Borkow, the heat-treatment method does not introduce additional chemicals; it is also very simple. The efficacy was tested against three bacteria that are responsible for HAIs: *Staphylococcus aureus*, *Pseudomonas aeruginosa*, and *Streptococcus pneumoniae*. Prevention of transmission is particularly important for pathogens where treatment options with antibiotics are more limited, and these three microbes are listed as serious threats by the CDC in their 2019 “Antibiotics Resistance Threats in the United States” [15]. Our results show that the Cu_2_O-treated facemask materials killed all three strains rapidly, reducing the number of viable cells by an average of 99.9% in 30 min. 

Bacteria frequently impinge on surfaces through droplets of bacterial suspensions, for example, respiratory droplets on facemasks or clothing. Our principal aim here is to determine how the bacteria in these droplets are killed by Cu_2_O. Several candidates for the active species for the antimicrobial/antiviral action of copper species have been considered [16,17]. Proposed mechanisms include (1) direct oxidation or polarization or binding by the ions (e.g., to the cell membrane or DNA); (2) reaction of copper ions with superoxide/hydrogen peroxide to produce reactive oxygen species (ROS), known as Fenton-type reactions; and (3) loss of function after direct contact of the microbe with the Cu_2_O solid [9,18,19]. There is some conflicting evidence from different sources, and it is possible that the mechanism depends on the bacterial species and the conditions. Results from the current work show that drying the test droplet onto the active solid is the main method of killing, and the presence of dissolved Cu^+^ and copper chelators affect bacterial survival. 

## 2. Materials and Methods

### 2.1. Materials

**Figure 1:**
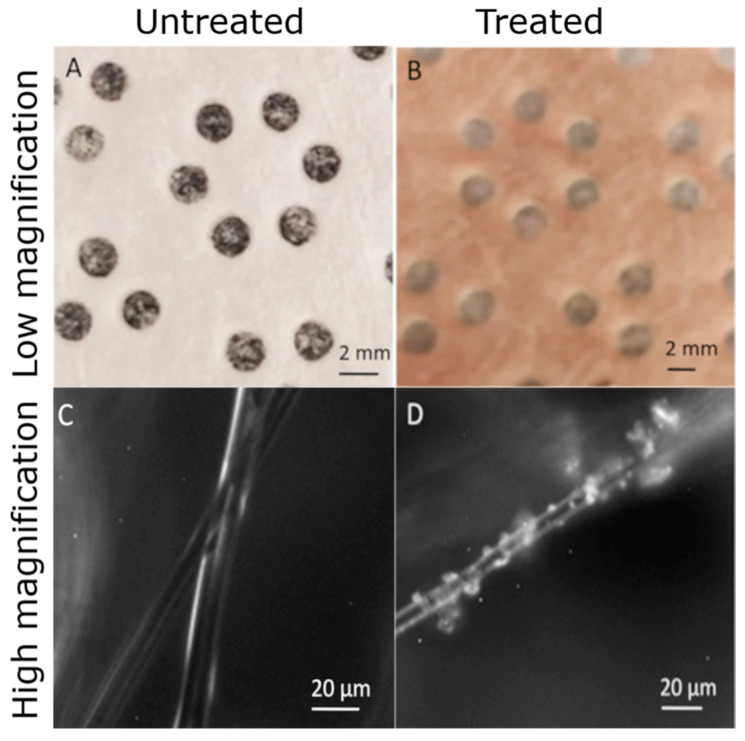
Light microscopy images of polypropylene materials at the macroscopic scale (**A**,**B**) and microscopic scale (**C**,**D**). The circular holes were in the original fabric and would not be present in all applications. After heat treatment, the attached 2 µm Cu_2_O particles impart a red-brown color on a macroscopic scale, and the particles can be seen on a microscopic scale.

Polypropylene (PP) nonwoven textiles (Kimtech Pure W4 Critical Task Dry Wipers) were purchased from Kimberly Clark. Within this fabric were holes that were approximately 1 mm in diameter (See Figure 1). Cu_2_O particles (Chem Copp HP III Type UltraFine-5) were purchased from the American Chemet Corporation. Metrics of the particle size and chemical composition are given in the Supporting Material, Tables S1 and S2. Diethyldithiocarbamic acid (ACS reagent grade Sodium diethyldithiocarbamate trihydrate) was purchased from Sigma-Aldrich (St. Louis, MO, USA). Bicinchoninic Acid (Bicinchoninic Acid Disodium Salt > 98% purity) was purchased from Tokyo Chemical Industry (Tokyo, Japan). One hundred percent ethanol (EtOH ACS grade) was purchased from VWR. Deionized (DI) water was from a Milli-Q Reference (MilliporeSigma) water purification system. 

### 2.2. Treatment of Polypropylene Fabric 

Particles were attached via heat treatment in a procedure similar to that described by Galante et al. [20] for preparing hydrophobic fabrics. One-inch diameter polypropylene textile samples were prepared using a punch. The as-received meltblown, nonwoven polypropylene was hydrophilic and was found to contain a surfactant. This surfactant and other ethanol-soluble contaminants were removed by soaking in 200-proof ethanol and then sonication in ethanol for 30 s. After further rinsing in ethanol, the fabric was left to dry in a laminar flow. At this point, the fabric was hydrophobic and suitable for the imbibition of Cu_2_O in ethanol suspensions. Twenty mg/mL of Cu_2_O in an ethanol suspension was mixed by sonication for 5 min, then vortexed, and then the fabric was immersed in the suspension and shaken on an Incubating Mini Shaker (VWR) at 400 rpm for 30 min before the fabric was removed from the suspension and dried. The dried fabric was heat treated in an oven at 130 °C for 30 min and then sonicated and rinsed in ethanol for 30 min to remove any loosely attached Cu_2_O particles. The material shown in Figure 1, which we subsequently call the “sample”, was finally dried in a laminar flow hood to prevent the deposition of unwanted particles. 

**Figure 2:**
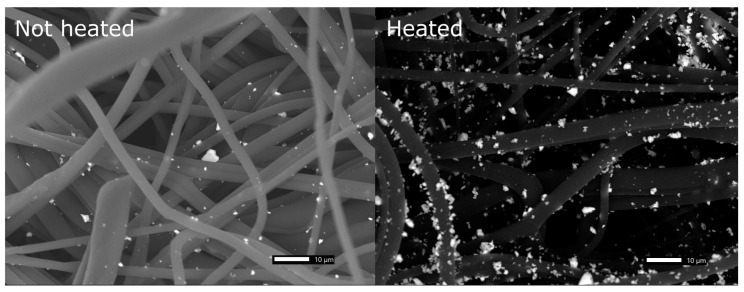
Scanning electron microscopy (SEM) images of polypropylene fabric treated with Cu_2_O particles. Left: with no heat treatment after particle attachment. Right: with heat treatment after particle attachment. Both samples were washed with ethanol, but the heat-treated sample retained a greater density of Cu_2_O particles.

It is important that the particles remain on the fibers throughout the usage period. To test the effect of heat treatment on Cu_2_O particle adhesion, 2 sets of treated polypropylene fabric were compared: one heat treated and one not. Three of each sample were weighed and then rinsed in ethanol and then sonicated in ethanol for 30 min and rinsed again to remove any loosely attached Cu_2_O particles. Without the heat treatment at 130 °C for 30 min, the mass increased by 2% ± 1%, whereas with the heat treatment, the mass increased by 11% ± 2%, i.e., about 5 times as much mass of particles was retained if the fabric was heat treated. Figure 2 is a set of scanning electron microscopy (SEM) images that also show the increase in particle retention. The mass data (*p* = 0.008) and images demonstrated the superior adhesion obtained by heat treatment, and so all further experiments were performed on heat-treated samples. All raw data is shown in Tables S3–S5.

### 2.3. Antibacterial Assay

*Microbial Strains.* The microbial strains employed in this study were a methicillin-resistant S. *aureus* (MRSA) MA43300, obtained from the Danville Community Hospital (Danville, VA), *P. aeruginosa* strain DSM-9644, and S. *pneumoniae* strain ATCC no. 6301. 

*Growth of Microbial Strains.* MRSA and P. *aeruginosa* were grown in 5 mL of Tryptic Soy Broth (TSB, BD, Sparks, MD), and S. *pneumoniae* was grown in 5 mL of Todd-Hewitt Broth (THB, Sigma-Aldrich, Saint Louis, MO, USA) to mid-exponential phase at 37 °C with aeration (60 rpm). Following growth, cell purity was identified by streaking onto Tryptic Soy Agar (TSA, BD) or Tryptic Soy Agar with 5% blood (TSAB, BD) and incubating at 37 °C for 24 h. Cells were only used if the size and shape of the colonies were consistent with known features of that strain; sample images of colonies are shown in Figure S1. 

*Preparation of Microbial Strains for Antimicrobial Testing*. Grown cells were separated from the growth medium by centrifugation at 5000 relative centrifugal force (rcf; =5000 g) for 5 min. The supernatant was discarded, and the cells were suspended in 5 mL of sterile phosphate-buffered saline (PBS) by vortexing for 60 s. Those suspensions were centrifuged (5000 rcf for 5 min), the supernatant was discarded, and washed cells were suspended in 5 mL of sterile PBS by vortexing for 60 s a second time. The number of colony-forming units (CFU)/mL of each washed suspension was measured by spreading 0.1 mL (in triplicate) of serial dilutions in PBS on TSA or TSAB plates. 

*Measurement of Cell Number.* The number of bacterial cells in a PBS suspension was measured as colony-forming units per mL suspension (CFU/mL). This measures the number of viable cells, i.e., those cells that were able to grow into a colony. A 10-fold dilution series was prepared for each PBS suspension, 0.1 mL of each dilution was spread on TSA or TSAB in triplicate, and colonies were counted after 24 h of incubation at 37 °C. If no colonies were present for the least dilution, we rounded the result up to one colony to enable a log transformation. We set this as the detection limit shown in the figures. Tables S6–S12 contain all CFU data.

*Measurement of Surface Killing.* For each bacterial strain, 5 lots of 2-μL droplets for a total volume of 10-µL of bacterial cell suspension in PBS suspension (totaling 10 μL on each sample) were placed on each of three individual Cu_2_O-treated or untreated polypropylene textiles. Multiple small droplets were used to decrease the time required for the droplet to evaporate. Since respiratory droplets are very small and will evaporate very quickly, we thought this would be a more realistic approximation of usage. Additionally, using several droplets allowed for a better sampling of the killing and can help control for inhomogeneities. Finally, a larger total volume provides better resolution of bacterial survival. After the desired time, each sample was transferred to a separate sterile 50 mL centrifuge tube containing 5 mL of sterile PBS, vortexed at the highest setting for 30 s, and sonicated for 1 min in a Branson model 12 ultrasonic cleaner (Shelton, CT, USA). In addition, 10 µL of the original suspension was diluted in 5 mL of PBS, and the cell density (CFU/mL) of this suspension was measured, as described above, to provide the input CFU (later used in Equation (1)). The room temperature was approximately 20 °C, and the relative humidity was approximately 25%. 

*Measurement of Solution Killing.* In addition to measuring surface killing, we also measured the antimicrobial activity of liquids, for example, liquids that contained ions extracted from the antimicrobial Cu_2_O. In these solution-killing experiments, 10 μL droplets of bacterial cells suspended in PBS solution were mixed with 90 μL of a challenge solution. After the desired time, 900 μL of PBS was added to the mixture, vortexed at the highest setting for 10 s, and then the CFU/mL of the suspension was measured as described above. 

*Examination of Ongoing Kill in CFU measurements*. There is a possibility that leachate from the Cu_2_O will kill or affect growth on the nutrient agar (“carryover”). Our results (See Supporting Material Table S12) showed that there was no significant effect of carryover. 

### 2.4. Durability of Treated Fabric to Airflow 

One important usage of polypropylene is in facemasks. For this particular use, the fabric is subject to period airflow arising from breathing, so we performed a test of the durability of the Cu_2_O coating subject to airflow. The apparatus consisted of an AC Infinity Axial 8025 Muffin Fan controlled by an Arduino Uno R3 that was programmed to turn the fan on and off at regular intervals (See Supplementary Materials, Apparatus, Figure S6). The interval was set to 5 s, which is approximately the time at which a human breathes [21]. The airflow was 0.62 m^3^/min, which was designed to be much greater than for normal human breath as a harsher test. Normal airflow is about 0.0057 m^3^/min for normal at-rest breathing and 0.04 m^3^/min during moderate exercise [21]. The treated mask materials were placed on this apparatus for up to 24 h, and the samples were weighed regularly to calculate the amount of Cu_2_O lost due to airflow. The experiment was conducted inside a laminar flow hood to prevent environmental contamination. Figure S3 shows that the 2 µm Cu_2_O particles were unaffected by airflow for up to 24 h, whereas there was a mass loss for the 5 µm particles (Figure S3), so all experiments in the results section utilized 2 µm particles. Human breath is humid, so we also checked the effect of humid air on the airflow (See Supplementary Materials, Apparatus, Figure S7). There was no effect for the 2 µm particles (Figure S4).

### 2.5. Effect of Leached Copper Ions on Bacterial Survival

To isolate the effect of copper ions on the antibacterial activity of Cu_2_O, chelating agents were used that are known to chelate copper, namely, sodium diethyldithiocarbamate trihydrate (DDTC, Sigma-Aldrich) and bicinchoninic acid (BCCA, Fischer Scientific, Hampton, FL, USA). To test the antibacterial activity of copper ions against bacteria and to test the effect of chelating agents against copper ions, a copper ion solution was prepared. A 200 mg/mL Cu_2_O in water suspension was vortexed for 5 min, sonicated for 5 min, and vortexed again for 5 min. The suspension was then centrifuged at 5000 rcf for 10 min, the supernatant collected, then centrifuged again at 5000 rcf for 10 min, and the supernatant collected again. The supernatant was finally filtered using a 1 μm filter (Whatman 1 μm PTFE syringe filter). The supernatant and chelating agent-supernatant mixtures were tested for their antibacterial activity by the solution-killing procedure described previously. Photographs of the steps appear in Figure S8.

### 2.6. Effect of Droplet Evaporation on Antimicrobial Effects

The rate of droplet evaporation was changed by placing the test solid plus the droplet in a humidity chamber (See Supplementary Materials, Apparatus, Figure S9) at 99% humidity (Extech Humidity Alert II digital hygrometer). The humidity was maintained by placing a moist paper towel inside the chamber. The surface killing was measured using the surface killing procedure described previously.

### 2.7. Statistics

Unless otherwise specified, all experimental conditions were done with triplicate samples. Statistical analysis consisted of ANOVA or a Student’s *t*-test, and *p*-values of about 0.05 or less were necessary conditions for a conclusion. Error bars in the figures refer to the 95% confidence interval for each particular sample condition.

## 3. Results and Discussion

### 3.1. Treated Polypropylene Fabric Rapidly Kills Bacteria Responsible for HAIs

**Figure 3:**
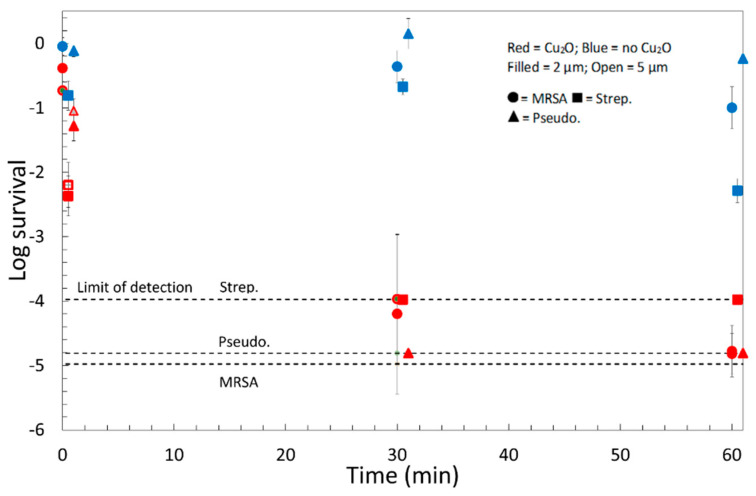
Time course of bacterial survival on polypropylene textiles with and without Cu_2_O-treatment. Note that the vertical axis is on a log_10_ scale. Data are shown for two particle sizes: 2 µm (filled symbols) and 5 µm (empty symbols). Circles are *P. aeruginosa*: squares are *S. pneumoniae,* and triangles are MRSA. Each data point represents the mean of the log of three independent measurements, and the error bars represent the 95% confidence interval for each condition. Data points for different organisms at the same time have been shifted horizontally for clarity. Data is tabulated in Tables S6–S8. The CFUs are reduced by 99.9% after 30 min for each organism on both the 2 µm and 5 µm samples.

The Cu_2_O-treated polypropylene fabric caused a dramatic reduction in the survival of all three bacteria tested, as shown in Figure 3. Our definition of survival is:

(1) $$\log\;\mathrm{survival}\equiv \mathrm{mean}\left[\log_{10}\left(\mathrm{sample}\;\mathrm{CFU}\right)\right]-\mathrm{mean}\left[\log_{10}\left(\mathrm{input}\;\mathrm{CFU}\right)\right]$$

where sample CFU refers to the CFU recovered from the test solid, and input CFU refers to the CFU obtained from the original test suspension. At zero time, the log survival would be 0 for an inactive solid if all the original bacteria were recovered from the solid by our extraction procedure. Because bacteria may die or divide over time, even for an inactive solid, we evaluate the performance of a coating by comparing the coated to the uncoated solid for the same exposure time using reduction:

(2) $$\log\;\mathrm{Reduction}\equiv \mathrm{mean}\left[\log_{10}\left(\mathrm{uncoated}\;\mathrm{CFU}\right)\right]-\mathrm{mean}\left[\log_{10}\left(\mathrm{coated}\;\mathrm{CFU}\right)\right]$$

A reduction of 99.9% in CFUs was seen for each strain by 30 min. By 1 h, the levels of both *S. pneumoniae* and *P. aeruginosa* had fallen below our detection limit. The data in Figure 3 contains three factors, the treatment, the time, and the organism, and was analyzed by analysis of variance (ANOVA) (see factors and *p*-values in Table S13). The important result is that the cross-term between time and treatment is significant (*p* = 6 × 10^−6^), which shows that the Cu_2_O-polypropylene results in a decreased lifetime of the bacteria. There was also a difference in the susceptibility of various organisms, which we believe is an artifact of the difference in the limit of detection of *S. pneumoniae*.

The particle size of cuprous oxide was unimportant (*p* = 0.8 for comparison between 2 µm and 5 µm particles), and from here, we explored only the properties of the 2 µm-Cu_2_O material because of its superior durability. Note that the Survival of *S. pneumoniae* on the untreated fabric decreased significantly between 30 and 60 min, which reduced our ability to resolve large reductions at 60 min.

In the remaining sections, we explore the important effects, such as drying and the presence of copper ions, that contribute to the death of the bacteria during our experiments.

### 3.2. Both Drying and the Copper Ion Concentration of the Test Droplet Affect the Survival of Bacteria

During the experiments described in Section 3.1**,** we noted that the droplets were partially dried after 30 min and fully dried by 1 h. We expect that the drying of the droplet is important, as previously observed [22,23], but clearly, drying alone does not determine killing here because far fewer bacteria died on untreated polypropylene. Drying could be an important part of the action of Cu_2_O through two effects: (a) drying reduces the drop volume, and (b) drying decreases the concentration of water available to the bacteria and concentrates non-volatile species. A reduction in drop volume brings the bacteria closer to the solid surface. If direct contact between the bacterium and the solid is required for an antimicrobial surface to be effective, then the transport time for bacteria to the surface is important. An evaporating droplet will provide a shorter average distance for both bacteria to travel to the surface and for leaching ions to reach the bacteria and will also provide convection to transport both species. Ultimately, when the droplet is in the final stages of drying, the suspended bacteria will be forced onto the solid by capillary action and will be progressively subject to increased concentrations of non-volatile solutes until those species precipitate. The behavior of copper ions is complex because they can dissolve from the coated test surface and re-precipitate during drying.

We performed an additional experiment in a humidity chamber at 99% humidity to control for the effect of drying. By raising the humidity to 99% and reducing airflow over the droplet, we found that the evaporation of the droplets was negligible over 1 h. To test the effect of dissolved copper ions, we also investigated the effect of a chelating agent that precipitates dissolved copper ions. We used the chelating agent, diethyldithiocarbamic acid (DDTC), which chelates both cupric (Cu^2+^) and cuprous (Cu^+^) ions [24,25]. DDTC was added to a bacterial suspension before placing the suspension droplet on the solid. In a separate experiment, we showed that DDTC did not kill the bacteria in suspension (See Supplementary Materials, Figure S5).

**Figure 4:**
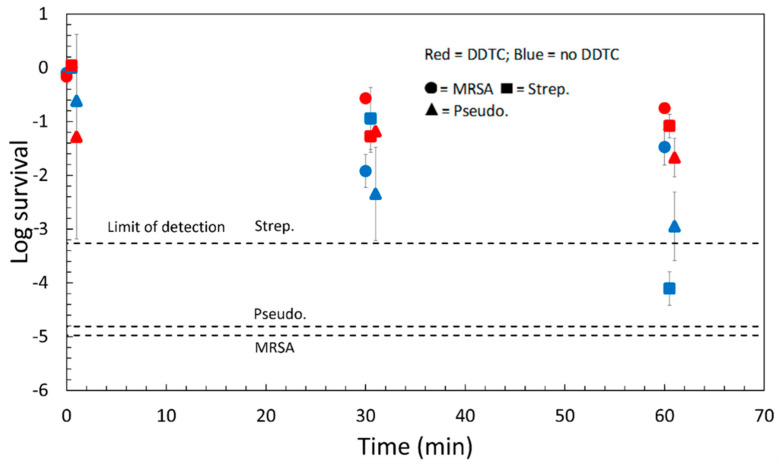
Time course of Survival on 2 µm Cu_2_O-treated polypropylene samples in the humidity chamber designed to reduce evaporation. Note that the vertical axis is on a log_10_ scale. Results are shown with or without 6 mM diethyldithiocarbamic acid (DDTC). Data tabulated in Table S9. The MRSA and Step. data at zero time are all at about log survival = 0. The CFU reduction with time for each strain was lower in the humidity chamber than in the biosafety cabinet. Overall, CFUs were greater in the humidity chamber and in the presence of DDTC.

Results for the Cu_2_O-coated polypropylene in the humidity chamber show a stark effect of droplet drying (Figure 4, *p* = 10^−9^), as seen by comparing Figure 3 and Figure 4. Survival data are tabulated in Table 1 for more convenient comparison, and ANOVA data is tabulated in Tables S14 and S15. In all cases, except *S. pneumoniae* at 60 min, the Survival was much greater (one to three orders of magnitude greater) when the droplet was not allowed to dry. At 60 min, the results are organism-dependent. Whereas the survival of all organisms is very low when evaporation is allowed (<0.001%), when drying is prevented, MRSA survival is ~3%, *P. aeruginosa* survival is ~0.1%, and only ~0.001% of *S. pneumoniae* survive in a droplet on the Cu_2_O surface. Droplet evaporation is very important in killing MRSA.

The chelating agent, DDTC, also has a large effect on killing: the survival of *P. aeruginosa* increases by 10× and *S. pneumoniae* increases by 1000× in the presence of DDTC. The Cu_2_O surface is very ineffective at killing organisms if there is no drying and a very low concentration of free copper ions. By removing the effect of drying, we have shown that dissolved copper ions make an important contribution to killing bacteria by the surface coating. An ANOVA (results in Table S14) showed that the chelator had a very large effect on the time course of killing (*p* = 3 × 10^−7^). A two-factor ANOVA was additionally run to assess the effect of time on the CFUs of humidity chamber samples with DDTC to see if time was significant when a chelator was introduced. The *p*-value for the time factor was 0.9, which suggests that time was not significant for the samples containing DDTC. One would expect that in the presence of an antimicrobial, survival would decrease with time (e.g., Figure 4). The lack of time-dependence with no drying and little free copper suggests that there are no important killing mechanisms beyond free dissolved copper species and drying onto the Cu_2_O surface.

### 3.3. Chelating Agents “Neutralize” Copper Ions

**Figure 5:**
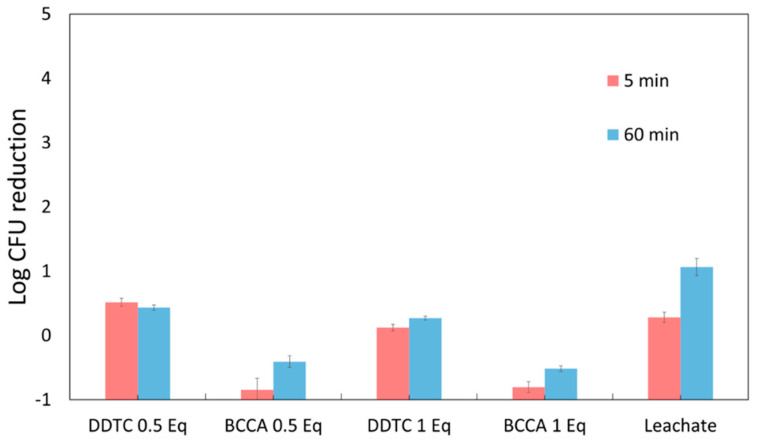
The activity of Cu_2_O leachate on MRSA in suspensions containing both leachate and suspended MRSA. 0.5 Eq. means that the amount of the chelating agent was 0.5 of the amount needed to chelate all the copper ions, etc. After 60 min, the leachate achieved a 1.1 log reduction from control *(p* = 0.006), and solutions containing DDTC and BCCA protected MRSA from the leached ions (*p* = 0.013 for DDTC and *p* = 0.007 BCCA for pairwise comparison with a 1-way ANOVA on the 60 min data using treatment as the sole factor).

**Figure 6:**
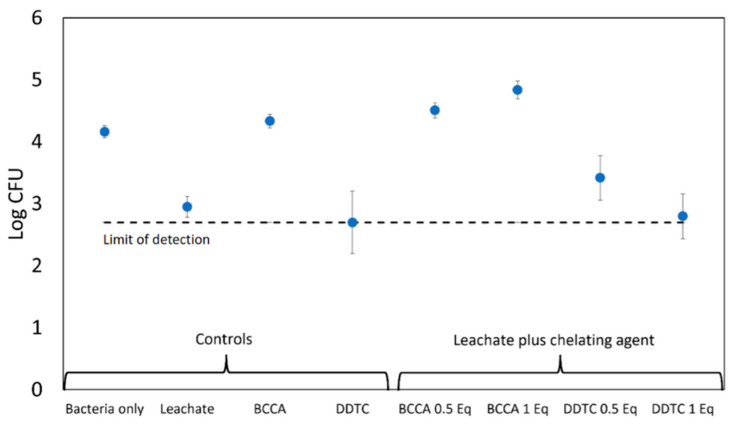
The activity of Cu_2_O leachate on MRSA in drying droplets containing both leachate and suspended MRSA. 0.5 Eq. means that the concentration of the chelating agent was 0.5 of the amount needed to chelate the copper ions, etc. DDTC becomes toxic when dried. BCCA maintains its protective effect against copper ions. Blue circles represent the average of three independent points and the error bars represent 95% confidence intervals for each condition.

The previous section indicated the importance of drying onto the solid and dissolved copper ions in the role of solid Cu_2_O particle activity against bacteria. To isolate the effects of copper species leached from Cu_2_O from the killing effect of the original Cu_2_O particles, we also report experiments of the antimicrobial activity of copper ion *solutions* where there is no solid Cu_2_O present. Suspensions of 1 µm Cu_2_O particles were mixed with water for 1.0 h, and then the suspension was filtered and centrifuged (Section 2.5) to remove solids. Further tests were performed on the supernatant, which we will refer to as the “leachate” because ions that leach from the Cu_2_O are in this liquid. Again, following the previous section, we know that drying may be important, and given that drying occurs in most applications, we allowed drying. Two chelating agents were used, DDTC, which chelates and precipitates both cupric and cuprous ions, and bicinchoninic acid (BCCA), which selectively chelates Cu^+^. Each was used at 0.01 M, which is about 10^5^× the solubility of Cu_2_O in water at pH 7.4 [26] to cause a dramatic reduction in copper ion solubility. The BCCA-Cu^+^ complex remains soluble, as is evident from the purple color of the solution. The experiment consisted of mixing leachate with bacterial suspension and sampling CFUs after 5 and 60 min and was performed on MRSA only. Results are shown in Figure 5. To study the effect of the drying state, 10 µL droplets of these mixtures were placed on inactive solid, polypropylene nonwoven fibers and allowed to dry for 30 min. Results are shown in Figure 6. This allows a test of the efficacy of drying without the presence of the Cu_2_O solid.

The results (Figure 5) show that leached copper species alone kill MRSA, as the supernatant reduced the MRSA CFUs by 1.1 log after 1 h of contact in solution. The killing effect was small after only 5 min of exposure, so results at 5 min will not be considered further. When the supernatant was mixed with a chelating agent before mixing in the bacteria, the log reduction decreased, which suggests that chelating agents have a protective effect against dissolved copper ions. This effect was observed whether the chelated ions were precipitated (for DDTC) or remained in solution (for BCCA). This is consistent with the results in the humidity chamber: about 1 log of killing also occurred when a chelator was present, and there was no drying to force the bacteria onto the solid. The BCCA results also show that when dissolved Cu^+^ is bound in chelated form, the leachate loses its antibacterial activity even when still in solution. This shows free Cu^+^ ions are the primary and perhaps the only ions in solution that are toxic to MRSA.

We found earlier that drying affects the ability of Cu_2_O to kill bacteria (Table 1), so the effect of the chelator was repeated in a drying droplet but without solid Cu_2_O present. To achieve this, we mixed Cu_2_O leachate with chelating agents, then added bacteria, then deposited 10 μL of three component mixture on polypropylene fibers and left the droplet for 30 min on the polypropylene fabric, after which the droplet was dry. We hypothesized that the droplet drying would concentrate the copper ions and, thereby, drying would promote killing and that the chelating agents would hinder this killing.

We were not surprised that the leachate killed the bacteria in the drying solution (*p* = 0.005), but there was only about 1.2 log of killing, which was about the same as for the leachate in suspension (Figure 5). So, drying did not have a big effect on the action of the dissolved copper species. This compares with the 3 logs of killing for the droplet that was dried directly in the presence of the Cu_2_O particles. Although dissolved copper ions kill about 94% of the MRSA, the 99.9% (3-log) killing only occurs when the bacterial suspension is dried onto the solid Cu_2_O particles.

To our surprise, we found that DDTC kills bacteria when the suspension dries. Therefore, DDTC is antimicrobial in concentrated form. This is a warning that when a chemical is dried and concentrated, it can be toxic to bacteria, even if it is not toxic in solution. This could easily cause confusion in drying droplet assays. The effect of DDTC on dissolved copper ions in drying droplets was, therefore, not considered further.

The results for BCCA were more interesting (Figure 6). BCCA alone in a drying droplet did not affect survival, so we were free to investigate its effect on the drying of copper leachate. Increasing amounts of BCCA increased survival. A linear regression analysis on the effect of BCCA concentration on log CFUs yielded a slope of 2.0 ± 0.6, which indicates that increasing amounts of BCCA protect the cells against even the potentially more concentrated Cu^+^ in a drying droplet. This tells us that Cu^+^ is an active dissolved species for Cu_2_O and that BCCA provides protection to MRSA against Cu^+^. Chelated but still soluble Cu^+^ is inactive.

We comment that the limited effect of dissolved Cu^+^ may be due to its low solubility in water or PBS (the medium used here). As the droplet dries, a buildup in Cu^+^ concentration will be limited by precipitation if the solution precipitation is in equilibrium. A more soluble species that exists in the original droplet may become highly concentrated and be antimicrobial at high concentrations that are not accessible to Cu^+^ in the conditions studied here. Our experiments do not exclude the possibility that it is the reprecipitated forms of dissolved copper that are the active species in the drying droplet.

Finally, we note that silver particles or silver chloride are often used as an active agent in antimicrobial coatings, clothing, or formulations. For silver, many mechanisms have been proposed and studied, and they are similar to those proposed for copper: silver ion uptake by cells, generation of reactive oxygen species, and direct contact with silver particles [27,28,29]. Our results show that for copper-based surfaces, surface contact is necessary to facilitate rapid killing; it is not clear whether contact is necessary for the action of silver.

## 4. Conclusions

The Cu_2_O-fabric rapidly kills three bacterial species (*S. aureus*, *P. aeruginosa*, *S. pneumoniae*) that are responsible for hospital-acquired infections: after 30 min, a typical CFU reduction is 99.9% compared to the uncoated material. The antimicrobial heat-treated Cu_2_O fabric may find application in reducing infection from equipment such as facemasks that are made from polypropylene. A similar heat treatment may be useful for other fibers that have a softening temperature.

Drying the test bacterial suspension droplet greatly diminishes the survival of the bacteria on the Cu_2_O-treated fabric: it is the combination of the Cu_2_O antimicrobial and drying that is effective. Furthermore, the main antimicrobial agent is the solid Cu_2_O itself. There is a significant, but much weaker, killing from Cu^+^ ions that leach from the Cu_2_O (94% of MRSA). Other species that dissolve from solid Cu_2_O are unimportant.

## Figures and Tables

**Table 1:** Comparison of Survival in an open container (Evap.) and humidity chamber (No Evap.).

Organism	Time (min)	Survival	
		Evap.	No. Evap.
MRSA	0	0.41	0.80
	30	0.00006	0.012
	60	0.00001	0.03
Strep	0	0.004	1.0
	30	0.0001 (<)	0.11
	60	0.0001 (<)	0.00008
Pseudomonas	0	0.052	0.24
	30	0.00001 (<)	0.005
	60	0.00001 (<)	0.001

< indicates below detection level; for MRSA and Pseudomonas, survival was much lower in the open container than in the humidity chamber.

## Data Availability

Data is contained in supporting information.

## Supplementary Materials

The following supporting information can be downloaded at: https://www.mdpi.com/article/10.3390/antibiotics11111633/s1, Figure S1: Photographs showing typical bacterial colonies of each organism; Figure S2: SEM image of 2 µm Cu_2_O particles; Figure S3: Effect of 0.62 m^3^/min airflow for 24 h on loss of Cu_2_O from polypropylene; Figure S4: Effect of humidity and airflow on mask durability after 24 h of airflow; Figure S5: CFU reduction by DDTC for cells in suspension; Figure S6: Photograph and circuit diagram of apparatus to provide airflow to simulate human breathing on a facemask; Figure S7: Photograph of apparatus for airflow experiments at controlled humidity; Figure S8: Photographs of the chelation of copper species by DDTC Figure S9: Photograph of the apparatus for controlling humidity during CFU measurements; Table S1: Cuprous oxide particle size distribution; Table S2: Cuprous oxide purity; Table S3: Weight% loss of Cu_2_O by washing of Cu_2_O-impregnated PP textiles; Table S4: Weight% loss of Cu_2_O by airflow durability testing against Cu_2_O-impregnated PP textiles; Table S5: Weight% loss of added Cu_2_O by airflow durability testing Cu_2_O-impregnated PP textiles in a humidity chamber; Table S6: Bacterial CFU data of MRSA on Cu_2_O-impregnated PP and control PP; Table S7: Bacterial CFU data of Pseudomonas on Cu_2_O-impregnated PP and control PP; Table S8: Bacterial CFU data of Strep on Cu_2_O-impregnated PP and control PP; Table S9: CFU data of bacterial test droplet on surfaces in a humidity chamber with and without a chelating agent (DDTC); Table S10: CFU data of cuprous ions and chelated cuprous ion solution killing; Table S11: CFU data of cuprous ions and chelated cuprous ion drying droplet killing; Table S12: Examination of ongoing kill on the nutrient agar plate; Table S13: Factors and *p*-values for ANOVA of Cu_2_O-treated polypropylene for the data in Figure 4; Table S14: ANOVA of humidity chamber CFU data; Table S15: ANOVA of humidity chamber and open container CFU data.
