# Supplementary material for: Mechanism and Efficacy of Cu2O-Treated Fabric
Source: Antibiotics (Basel). 2022 Nov 16;11(11):1633. doi: 10.3390/antibiotics11111633 (PMC9686972; doi:10.3390/antibiotics11111633)
Supplement: Supplementary file 1 [file antibiotics-11-01633-s001.zip › antibiotics-2013007-supplementary.pdf]

Supplementary Materials

Mechanism and Efficacy of Cu_2O-Treated Fabric

Zachary Benmamoun, Trent Wyhopen, You Li, and William A. Ducker*

*Corresponding Authors' Email: wducker@vt.edu

Dept. of Chemical Engineering and Center for Soft Matter and Biological Physics, Virginia Tech, Blacksburg, Virginia, 24061, USA

S1. Particle Size and Chemical purity

Lot U1957 (5 µm)		Lot 123942 (2 µm)	
Mean size (µm)	5.5	Mean size (µm)	2.0
Median size (µm)	5.0	Median size (µm)	1.6
Mode size (µm)	5.5	Mode size (µm)	1.2
St dev (µm)	3.1	St dev (µm)	1.4

Table S1. Cuprous oxide particle size distribution

Note that the particles that we label as 1 µm had a mean size of 2.0 µm and are labelled as 2 µm in the paper.

Lot U1957 (5 µm)		Lot 123942 (2 µm)	
Cuprous oxide %	96.2	Cuprous oxide %	94.6
Cupric oxide %	2.1	Cupric oxide %	3.6
Metallic Cu	0.7	Metallic Cu	1.0
Lead ppm	61	Lead ppm	125
Cadmium ppm	0	Cadmium ppm	0
Arsenic ppm	0	Arsenic ppm	3

Table S2. Cuprous oxide purity

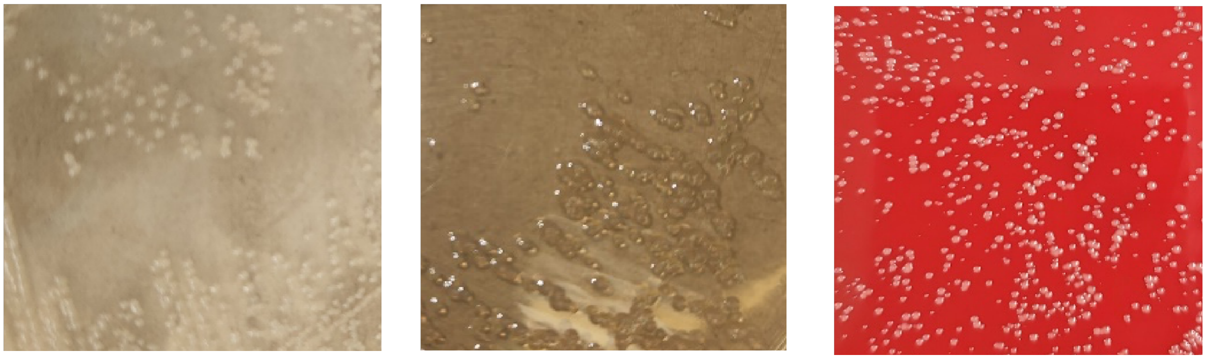

MRSA colonies on TSA *P. aeruginosa* colonies on TSA *S. pneumoniae* colonies on TSAB

Figure S1. Photographs showing typical bacterial colonies of each organism.

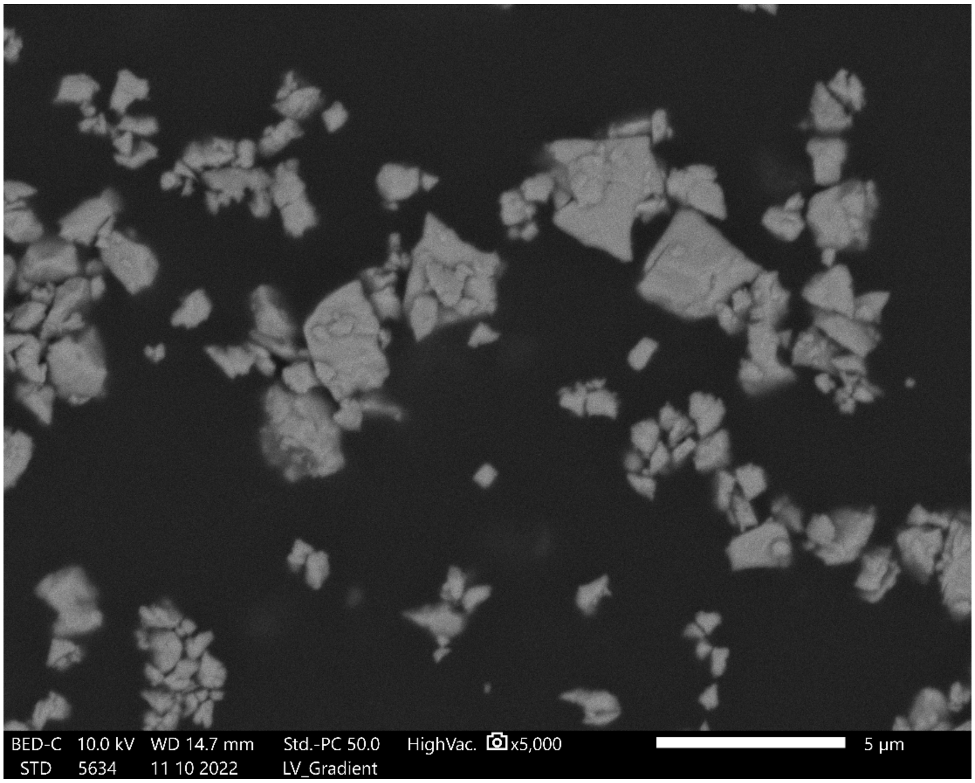

Figure S2. SEM image of 2 µm Cu_2O particles

S2. Data Tables

Treatment	Time (min)	% Cu_2O lost by washing
Unheated samples	5	58.2
	10	62.9
	15	61.6
	30	77.8
	45	85.3
Heated samples	5	40.2
	10	32.3
	15	51.1
	30	29.6
	45	29.8

Table S3. Weight % loss of Cu_2O by washing of Cu_2O-impregnated PP textiles. Heat treated and not heat treated are compared.

Material	Time	Average % loss
2 µm Cu_2O on PP	0	0
	15	0.05
	30	-0.7
	45	-0.9
	60	0.09
	75	-0.6
	90	-0.4
	105	-0.2
	1440	5.2

5 µm Cu_2O on PP	0	0
	15	2.4
	30	4.0
	45	8.3
	60	10.6
	75	13.3
	90	11.7
	105	13.4
	1440	16.1

Table S4. Weight % loss of Cu_2O by airflow durability testing against Cu_2O-impregnated PP textiles.

Material	Time (min)	Average % loss
2 µm Cu_2O on PP	0	0
	1440	5.5
5 µm Cu_2O on PP	0	0
	1440	8.3

Table S5. Weight % loss of added Cu_2O by airflow durability testing against Cu_2O-impregnated PP textiles in humidity chamber.

Material	Time (min)	CFU	Log CFU
Nil (input bacteria control)	0	4x10^{6}	6.7
		5x10^{6}	
		6x10^{6}	
Polypropylene (PP)	0	4x10^{6}	6.6
		6x10^{6}	
		4x10^{6}	
	5	3x10^{6}	6.5
		3x10^{6}	
		3x10^{6}	
	10	3x10^{6}	6.4
		2x10^{6}	
		3x10^{6}	
	30	1x10^{6}	6.4
		4x10^{6}	
		2x10^{6}	
	60	1x10^{6}	5.7
		3x10^{5}	
		4x10^{5}	

1 µm Cu_2O on PP	0	$2x10^6$	6.3
		$2x10^6$	
		$2x10^6$	
	5	$4x10^4$	5.4
		$5x10^5$	
		$2x10^5$	
	10	$6x10^4$	4.8
		$7x10^4$	
		$4x10^4$	
	30	$1x10^2$	3.3
		$5x10^3$	
		50	
	60	50	2
		50	
		$2x10^2$	
5 µm Cu_2O on PP	0	$1x10^6$	5.9
		$7x10^5$	
		$9x10^5$	
	5	$3x10^5$	5.6
		$6x10^5$	
		$4x10^5$	
	10	$5x10^5$	5.2
		$2x10^3$	
		$1x10^4$	
	30	$2x10^3$	3.0
		50	
		$1x10^3$	
	60	$2x10^2$	1.9
		50	
		50	

Table S6. Bacterial CFU data of MRSA on Cu_2O – impregnated PP and control PP.

Material	Time (min)	CFU	Log CFU
Nil (input bacteria control)	0	$3x10^6$	6.5
		$3x10^6$	
		$3x10^6$	
Polypropylene (PP)	0	$3x10^6$	6.4
		$2x10^6$	
		$2x10^6$	
	30	$8x10^6$	6.7
		$4x10^6$	
		$3x10^6$	
	60	$2x10^6$	6.3
		$2x10^6$	

		$2x10^{6}$	
1 µm Cu_2O on PP	0	$2x10^{5}$	5.2
		$1x10^{5}$	
		$2x10^{5}$	
	30	<	<
	60	<	<
5 µm Cu_2O on PP	0	$3x10^{5}$	5.5
		$4x10^{5}$	
		$2x10^{5}$	
	30	<	<
	60	<	<

Table S7. Bacterial CFU data of Pseudomonas on Cu_2O – impregnated PP and control PP. < means that zero colonies were observed.

Material	Time (min)	CFU	Log CFU
Nil (input bacteria control)	0	$4x10^{5}$	6.7
		$5x10^{5}$	
		$5x10^{5}$	
	0	$9x10^{4}$	4.9
Polypropylene (PP)		$1x10^{5}$	
		$5x10^{4}$	
	30	$1x10^{5}$	5.0
		$8x10^{4}$	
		$1x10^{5}$	
	60	$3x10^{3}$	3.4
		$3x10^{3}$	
		$2x10^{3}$	
1 µm Cu_2O on PP	0	$2x10^{3}$	3.4
		$4x10^{4}$	
		$1x10^{3}$	
	30	<	<
		<	
		<	
	60	<	<
		<	
		<	
5 µm Cu_2O on PP	0	$2x10^{3}$	3.5
		$6x10^{3}$	
		$3x10^{3}$	
	30	<	<
		<	
		<	
	60	<	<

		<	
		<	

Table S8. Bacterial CFU data of Strep on Cu_2O – impregnated PP and control PP.

Bacterium	Material	Time (min)	CFU	Log CFU
MRSA	1 µm Cu_2O on PP	0	$4x10^6$	6.6
			$4x10^6$	
			$4x10^6$	
		30	$9x10^4$	4.8
			$3x10^4$	
			$8x10^4$	
		60	$2x10^5$	5.3
			$2x10^5$	
			$7x10^4$	
	1 µm Cu_2O on PP with DDTC	0	$4x10^6$	6.5
			$3x10^6$	
			$3x10^6$	
		30	$1x10^6$	6.1
			$1x10^6$	
			$1x10^6$	
		60	$9x10^5$	5.9
			$9x10^5$	
			$8x10^5$	
Pseudomonas aeruginosa	1 µm Cu_2O on PP	0	$2x10^5$	5.3
			$1x10^5$	
			$2x10^5$	
		30	$3x10^3$	4.6
			$1x10^5$	
			$1x10^4$	
		60	$2x10^3$	3.8
			$2x10^4$	
			$1x10^3$	
	1 µm Cu_2O on PP with DDTC	0	$2x10^3$	5.3
			$1x10^3$	
			$2x10^3$	
		30	$2x10^5$	5.3
			$3x10^5$	
			$2x10^5$	
		60	$1x10^5$	4.9
			$3x10^4$	
			$8x10^4$	
Streptococcus pneumoniae	1 µm Cu_2O on PP	0	9´10^5	6.0
			$1x10^6$	

			$9x10^5$	
		30	$2x10^5$	5.2
			$2x10^5$	
			$3x10^4$	
		60	<	1.9
			<	
			$2x10^2$	
	1 µm Cu_2O on PP with DDTC	0	1´10^6	6.0
			$9x10^5$	
			$1x10^6$	
		30	$9x10^4$	4.7
			$4x10^4$	
			$3x10^4$	
		60	$1x10^5$	4.9
			$5x10^4$	
			$9x10^4$	

Table S9. CFU data of bacterial test droplet on surface in humidity chamber with and without chelating agent (DDTC).

Material	Time	CFU	Log CFU
Nil (input bacteria)	5	$3x10^5$	5.5
		$3x10^5$	
		$4x10^5$	
	60	$3x10^5$	5.5
		$3x10^5$	
		$3x10^5$	
DDTC control	5	$2x10^5$	5.3
		$2x10^5$	
		$2x10^5$	
	60	$4x10^5$	5.6
		$4x10^5$	
		$4x10^5$	
BCCA control	5	$2x10^5$	5.1
		$1x10^5$	
		$2x10^5$	
	60	$4x10^5$	5.6
		$4x10^5$	
		$4x10^5$	
Cu_2O control	5	$3x10^5$	5.2
		$3x10^5$	
		$4x10^5$	
	60	$3x10^5$	4.4
		$3x10^5$	
		$3x10^5$	
½ DDTC	5	$5x10^4$	4.8
		$6x10^4$	

		$7x10^4$	
	60	$1x10^5$	5.2
		$2x10^5$	
		$2x10^5$	
½ BCCA	5	$8x10^5$	6.0
		$1x10^6$	
		$8x10^5$	
	60	$9x10^5$	6.0
		$9x10^5$	
		$1x10^6$	
Full DDTC	5	$1x10^5$	5.2
		$1x10^5$	
		$2x10^5$	
	60	$2x10^5$	5.3
		$2x10^5$	
		$2x10^5$	
Full BCCA	5	$8x10^5$	5.9
		$1x10^6$	
		$48x10^5$	
	60	$1x10^6$	6.1
		$1x10^6$	
		$1x10^6$	

Table S10. CFU data of cuprous ions and chelated cuprous ion solution killing.

Material	Time	CFU	Log CFU
Nil (input bacteria)	30	$4x10^4$	4.2
		$6x10^4$	
		$7x10^4$	
DDTC control	30	<	<
		<	
		<	
BCCA control	30	$2x10^4$	4.3
		$3x10^4$	
		$2x10^4$	
Cu_2O control	30	$2x10^3$	2.9
		$2x10^3$	
		$1x10^3$	
½ DDTC	30	$2x10^3$	3.4
		$2x10^3$	
		$8x10^3$	
½ BCCA	30	$3x10^4$	4.5
		$5x10^4$	
		$3x10^4$	
Full DDTC	30	$1x10^3$	2.8
		<	
		<	

Full BCCA	30	$5x10^4$	4.8
		$4x10^4$	
		$2x10^5$	

Table S11. CFU data of cuprous ions and chelated cuprous ion drying droplet killing.

Chemistry	Bacterium	CFU	Log CFU
No DDTC	MRSA	$3x10^6$	6.4
		$2x10^6$	
		$2x10^6$	
	Pseudomonas aeruginosa	$3x10^6$	6.4
		$3x10^6$	
		$3x10^6$	
	Streptococcus pneumoniae	$2x10^5$	6.0
		$2x10^5$	
		$5x10^3$	
DDTC	MRSA	$3x10^6$	6.5
		$3x10^6$	
		$4x10^6$	
	Pseudomonas aeruginosa	$2x10^6$	6.4
		$3x10^6$	
		$2x10^6$	
	Streptococcus pneumoniae	$5x10^4$	5.3
		$3x10^5$	
		$2x10^5$	

Table S12. Examination of ongoing kill on the nutrient agar plate. We considered the possibility that leachate from the Cu_2O kills or affects growth on the nutrient agar. To examine this effect, we immersed Cu_2O-coated fabric in PBS and then bacterial cells were added in suspension. Then we followed our normal procedure of vortexing and sonication and plating cells on the agar that was done in our CFU measurements. Two conditions were examined, one where the chelating agent, DDTC, was added before the cells were introduced and the other where no DDTC was added. Since we know that DDTC inhibits the action of Cu_2O, if there were an ongoing kill on the agar, then we would expect there to be more cells on the agar plate when the DDTC was present. Our results show no significant difference ($P = 0.36$ for effect of DDTC and $P = 0.54$ for combined effect of Organism and DDTC in 2-way ANOVA), which is consistent with no ongoing kill on the plate. A simple t-test for *S. pneumoniae* also showed no significant difference.

**Factor**	***p*-value**
Treatment (Cu_2O vs none)	4×10^{-23}
Time	7×10^{-18}
Organism	3×10^{-9}
Time x Treatment	6×10^{-6}
Time x Organism	1×10^{-2}
Organism x Treatment	5×10^{-2}

Table S13. Factors and *p*-values for ANOVA of Cu_2O-treated polypropylene for the data in Figure 3. Treatment factors are no treatment, 2 µm Cu_2O, and 5 µm Cu_2O. Response was log CFU.

Factors	*p*-value
Organism	2×10^{-7}
Time	2×10^{-7}
Time x Chelator	3×10^{-7}
Time x Organism	3×10^{-4}
Chelator (DDTC or none)	6×10^{-3}
Organism x Chelator	7×10^{-2}

Table S14. ANOVA of humidity chamber CFU data. Response was log CFU. The chelator has a significant effect on the time-course of killing.

Factors	*p*-value
Time	3 × 10-12
Evaporation	1×10^{-9}
Organism	5×10^{-4}
Time x Evaporation	4×10^{-1}
Organism x Evaporation	8×10^{-1}
Time x Organism	0.1

Table S15. ANOVA of humidity chamber and open container CFU data. Response was log CFU. Evaporation is significant, therefore drying significantly affects bacterial CFUs.

S3. Supporting Figures

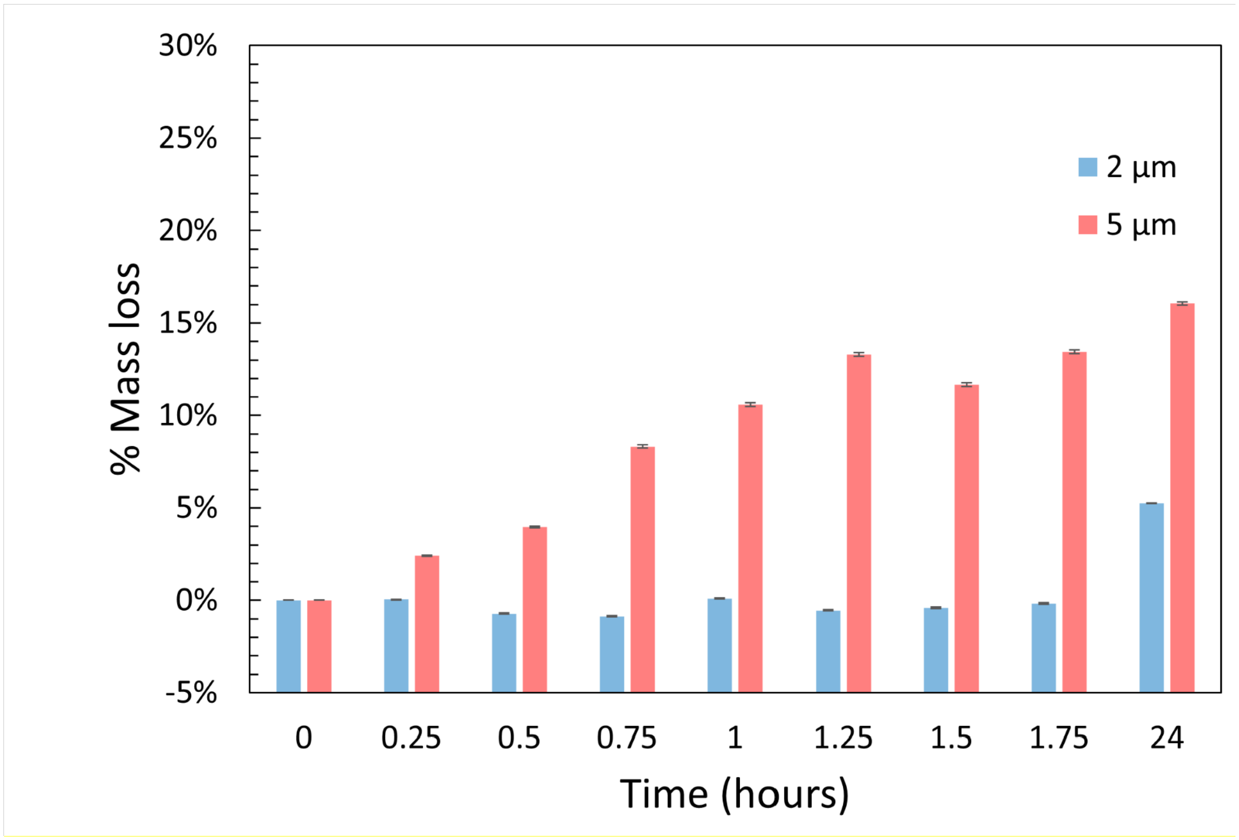

Figure S3. Effect of 0.62 m^3/min airflow for 24 h on loss of Cu_2O from polypropylene. The vertical axis is the mass of Cu_2O at the specified time divided by the initial mass of Cu_2O. The fabric made with 2 µm Cu_2O particles was more durable to airflow: it lost only 5.2% of the initial Cu_2O with 100´ the normal breathing air flow rate whereas 16% of the 5 µm particles were lost.

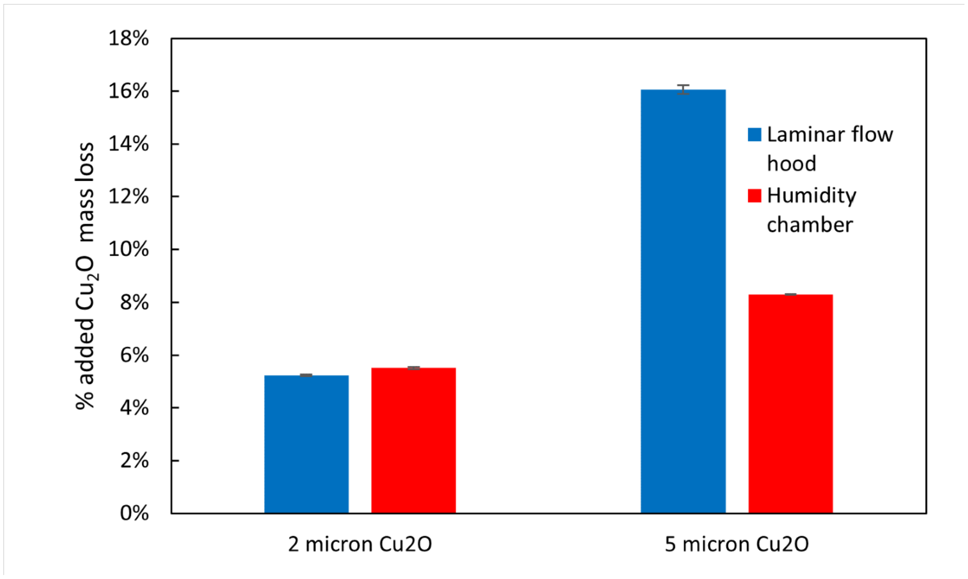

Figure S4. Effect of humidity and airflow on mask durability after 24 hours of airflow. There is little effect of humidity.

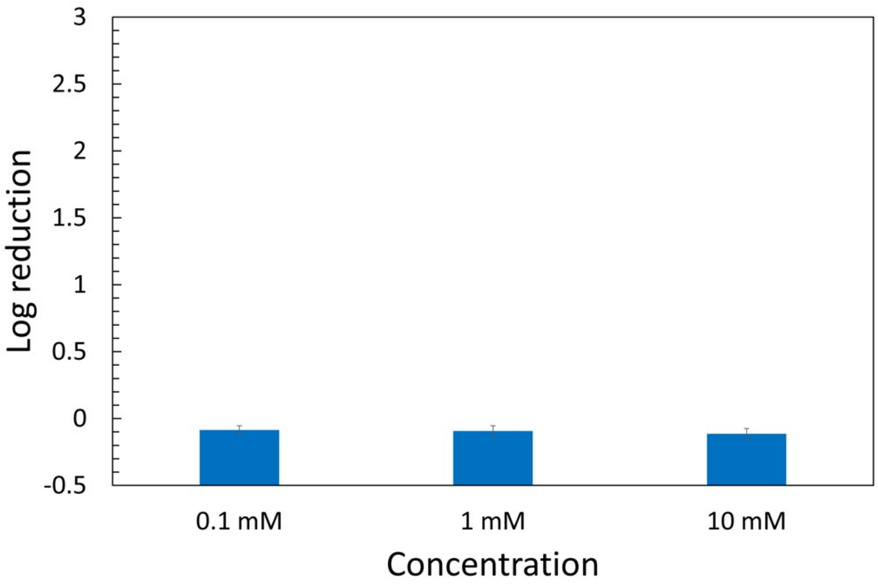

Figure S5. CFU reduction by DDTC for cells in suspension. Log Reduction of zero means that the DDTC did not kill any bacteria.

APPARATUS

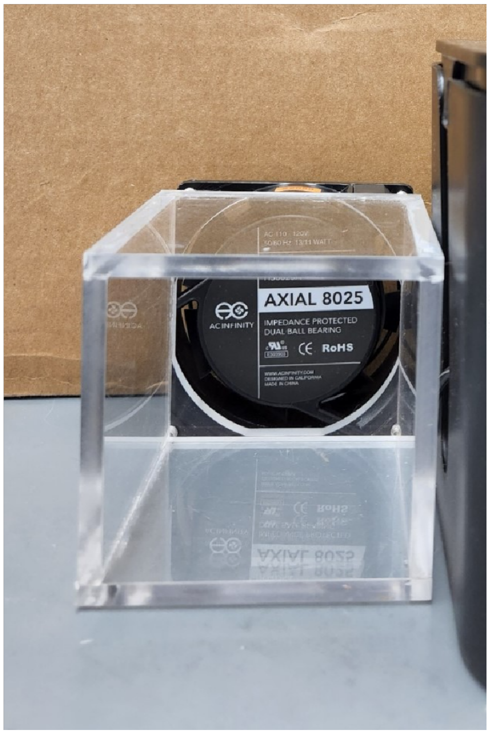

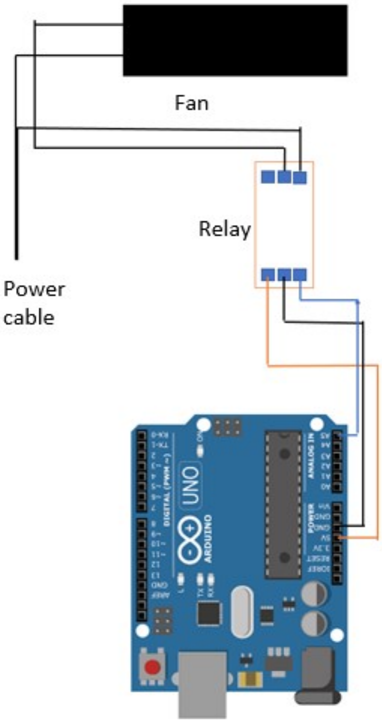

Figure S6. Photograph and circuit diagram of apparatus to provide airflow to simulate human breathing on a facemask. Note that the flow alternated between positive flow and off, there was no negative flow.

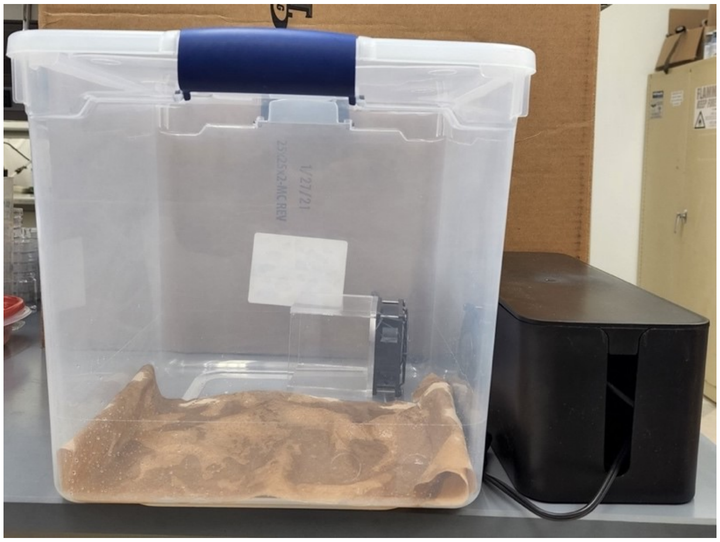

Figure S7. Photograph of apparatus for airflow experiments at controlled humidity.

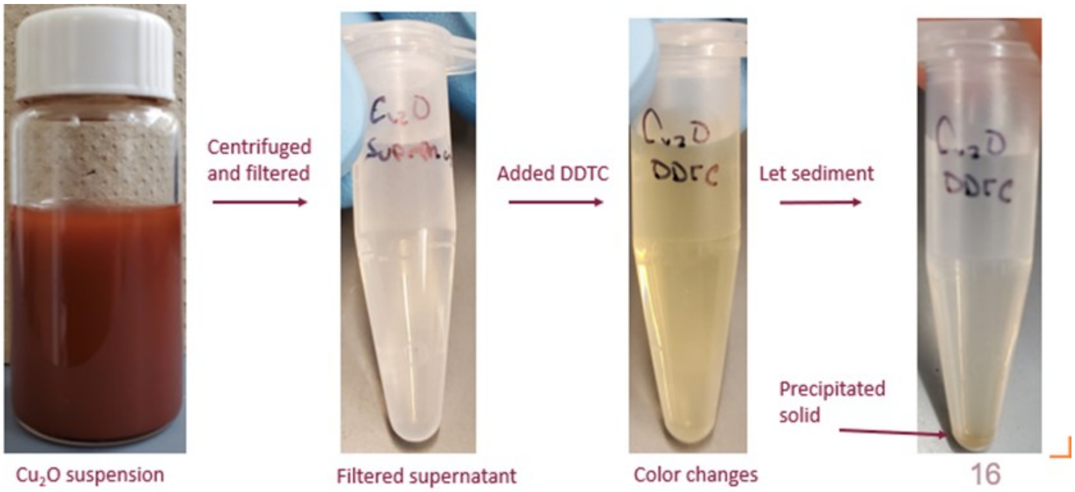

Figure S8. Photographs of chelation of copper species by DDTC. There are no cells for these photographs.

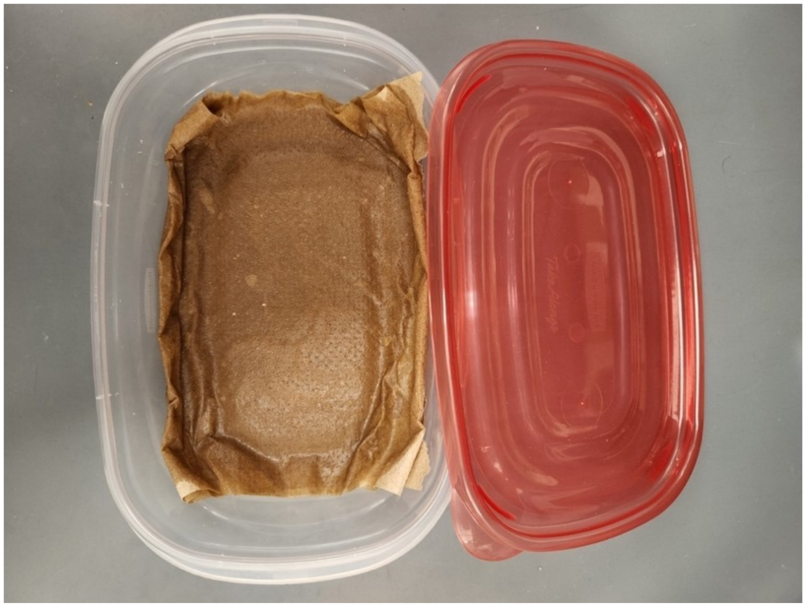

Figure S9. Photograph of apparatus for controlling humidity during CFU measurements.
